# Coprime dual-velocity encoding for extended velocity dynamic range in 4D flow magnetic resonance imaging

**DOI:** 10.1016/j.jocmr.2025.101871

**Published:** 2025-03-07

**Authors:** Marta Beghella Bartoli, Sara Boccalini, David Chechin, Loic Boussel, Philippe Douek, Damien Garcia, Monica Sigovan

**Affiliations:** a University of Lyon, CREATIS Laboratory, Lyon, France; b Department of Radiology, Hospices Civils de Lyon, Lyon, France; c Philips, Surenes, France

**Keywords:** 0000, 1111, 4D flow MRI, Dual-VENC, Flow phantom, Extended velocity dynamic range, VNR

## Abstract

**Background:**

In the field of cardiovascular imaging, four-dimensional (4D) flow cardiovascular magnetic resonance (CMR) provides non-invasive assessment of blood flow. Dual velocity encoding (dual-VENC) strategies have emerged to obtain quantitative information on both low and high blood flow velocities simultaneously. However, these strategies often encounter difficulties in coping with large velocity ranges. This work presents a dual-VENC 4D flow CMR sequence that utilizes the coprime rule to define the VENC ratio.

**Methods:**

A dual-VENC 4D flow CMR sequence and reconstruction algorithm were developed and validated *in vitro* at two different field strengths, using a flow phantom generating realistic complex flow patterns. A digital twin of the phantom allowed comparison of the MRI measurements with computational fluid dynamics (CFD) simulations. Three patients with different cardiac pathologies were scanned in order to evaluate the *in vivo* feasibility of the proposed method.

**Results:**

The results of the *in vitro* acquisitions demonstrated significant improvement in velocity-to-noise ratio (VNR) with respect to single-VENC acquisitions (110 ± 3%) and conventional dual-VENC de-aliasing approach (75 ± 3%). Furthermore, the effectiveness of aliasing correction was demonstrated even when both sets of images from the dual-VENC acquisition presented velocity aliasing artifacts. We observed a high degree of agreement between the measured and simulated velocity fields.

**Conclusion:**

The strength of this approach lies in the fact that, unlike the conventional de-aliasing method, no data is discarded. The final image is obtained by a weighted average of the *VENC*_*low*_ and *VENC*_*high*_ datasets. Consequently, setting the value of the *VENC*_*high*_ to prevent aliasing is no longer necessary, and higher VNR gains are possible

## Graphical abstract

**Figure:**
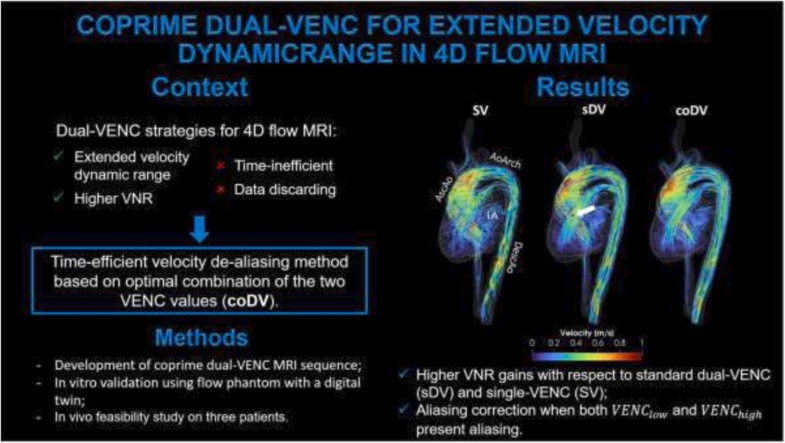

## 1 Introduction

Four-dimensional (4D) flow cardiovascular magnetic resonance (CMR) is a phase contrast (PC) technique that enables a comprehensive assessment of cardiovascular function by providing a quantitative volumetric description of blood flow throughout the cardiac cycle [1], [2], [3], [4]. 4D Flow MRI makes use of flow-encoding gradients to quantify the velocity of complex blood flows. Consequently, velocity is proportional to the net phase accumulation of the MR signal and is thus inherently bounded by the velocity encoding parameter (VENC), limiting the dynamic range of measured velocities to the range of − *VENC* to *VENC*. Being a volumetric method, complex blood flows with low velocities in veins and high velocities in arteries occur within the same measurement volume even in healthy subjects. Therefore, choosing the appropriate VENC value is crucial, and its selection represents a fundamental trade-off in 4D Flow CMR. Setting VENC too low leads to velocity aliasing, or phase wrapping, for velocities beyond ± VENC, due to the limited dynamic range. On the other hand, setting VENC high leads to noisy inaccurate measurements of slow flow regions [5], [6]. The ideal measurement should provide good velocity-to-noise ratios (VNR) for precise measurements of slow flow and an extended velocity dynamic range to avoid aliasing artifacts in fast flows.

Early works in weather radar signal processing addressed a different but related challenge, extending the velocity dynamic range while maintaining sufficient coverage at various distances. These efforts introduced methods such as pulse pairs with different sampling rates to extend the measured velocity dynamic range [7]. One such method involved the use of alternating pulse repetition frequencies (PRFs), where pulses are transmitted at different time intervals to mitigate aliasing. Since different PRFs result in different velocity limits, combining velocity measurements from the two PRFs can significantly extend the unambiguous velocity range [8] when the ratio between the two velocities can be expressed as the ratio of coprime (or relatively prime) integers [9]. Two integers are said to be coprime (or relatively prime) if they have no common positive factors other than 1. The velocity dynamic range can be extended p-fold (with p being an integer) by determining the extended velocity based on the relationship between the low and high PRF values. Typically, a ratio of p/(p+1) is chosen between the low and the high PRF to ensure that the two integers are coprime, where p is the extension factor governing the increase in the extended range. When the ratio p/(p+1) is close to 1, indicating that the low and high unambiguous velocities are close, the velocity dynamic range can be extended markedly. Nevertheless, high extension factors degrade measurement accuracy, limiting their practical application. Therefore, extension factors of 2, 3, or 4 are recommended to balance velocity range and measurement quality [10]. Building on this, the dual-PRF method was further generalized for multiple-PRF by [11] for color Doppler echocardiography.

Similarly, extending the velocity dynamic range in PC MRI involves taking two or more measurements with different velocity limits, using techniques known as dual- or multi-VENC. However, the ratios chosen between the low to high velocities are generally low (≤0.5), thus the extension factor is 1 [6], [13], [12]. Standard dual-VENC-based velocity unwrapping utilizes the *VENC*_*high*_ data to detect phase jumps in the *VENC*_*low*_ data and to calculate the number of phase wraps that occurred. It then adds or subtracts the appropriate multiple of 2*π* in the selected voxels. This results in an image with the higher velocity-to-noise ratio (VNR) of the *VENC*_*low*_ acquisition and the velocity range of the *VENC*_*high*_ acquisition [6]. However, in the presence of pathology, standard dual-VENC unwrapping may be limited, as unexpectedly high velocities [14] can result in aliasing in the *VENC*_*high*_ data [6], [15]. In this context, using of larger extension factors (*p*≥2) appears advantageous. Notably, [16] reintroduced the concept of the p/(p+1) ratio and demonstrated the feasibility of using p factors of 2 and 3 to correct velocity aliasing for 2D phase-contrast MRI. An additional advantage of this method is that the true velocity does not need to be known precisely in advance, as aliasing is permitted for both VENCs. Recently, [17] proposed a correction algorithm for the initial implementation, and reported similar unwrapping performance compared to standard [6] and triconditional unwrapping methods [15]. However, these works were applied exclusively in 2D and did not include implementation of a dual-VENC sequence. In addition, one drawback of the proposed methods is the computation time, since velocity unwrapping involves a computationally expensive minimization algorithm. We sought to build on the aforementioned studies by introducing a 4D Flow MRI dual-VENC sequence that utilizes the coprime rule for the VENC ratio, enabling an extended velocity dynamic range. We introduce a time-efficient velocity unwrapping algorithm. We validate performance through *in vitro* experiments and demonstrate *in vivo* feasibility in patients with cardiovascular pathologies.

## 2 Methods

### 2.1 Dual-VENC pulse sequence

We implemented a dual-VENC 4D Flow CMR sequence on Philips systems. The sequence uses an interleaved 8-point velocity encoding. The first gradient echo measures the reference MR-signal phase, and the following three TRs apply velocity encoding gradients along the three orthogonal directions for *VENC*_*low*_. This scheme is then repeated in the following four segments, with the first moment of the bipolar gradients changing for *VENC*_*high*_ [13]. Six sets of phase difference images are obtained by subtracting the reference MR-signal phase from each of the six flow-sensitive MR-signal phases. The resulting data is three-directional velocity maps for *VENC*_*high*_ and *VENC*_*low*_. Echo-time (TE) and repetition-time (TR) of the dual-VENC sequence were consistent between encodings and computed as the minimum possible values given the *VENC*_*low*_ setting.

### 2.2 Proposed Dual-VENC reconstruction

#### 2.2.1 Velocity unwrapping

The dual-VENC based velocity unwrapping algorithm that we propose in this paper has been adapted from a fast numerical method for velocity aliasing correction initially developed for Doppler weather radar [7]. This method was generalized for ultrafast color Doppler using multiple pulse-repetition-frequency (PRF) emissions arranged in a series of staggered intervals [11]. In particular, dual-PRF results in two sets of velocity images with different dynamic ranges that alias differently. Likewise, our dual-VENC method produces two three-component velocity sets that alias distinctly. When combined, aliasing can be removed as described below.

When velocity aliasing is present, the expression that relates the unambiguous velocity (*Vu*) and the measured velocity (*Vm*) can be written as follows:

(1) $$Vu=Vm+2\;n_{N}\;VENC,$$

where *n*_*N*_ is the Nyquist number (with *n*_*N*_ ∈ *Z*). Measured velocities are aliased when *n*_*N*_ ≠ 0. When two sets of velocity images that alias differently are available, the Nyquist numbers can be estimated from the velocity differences. It has been shown [11] that it is advantageous to choose the two VENC values in terms of a ratio as a function of the integer *p*≥2:

(2) $$VENC_{low}=\frac{p}{p+1}\cdot VENC_{high}.$$

The unambiguous (i.e. alias-corrected) velocity can be resolved whenever its absolute value is less than the extended Nyquist velocity (*VENC*_*ext*_):

(3) $$VENC_{ext}=p\cdot VENC_{high}.$$

Unwrapping aliased velocities consists of finding the Nyquist numbers *n*_*Nhigh*_ and *n*_*Nlow*_ that satisfy:

(4) $$nint\left(\left(p+1\right)\frac{Vm_{low}-Vm_{high}}{pVENC_{high}}\right)=n_{Nhigh}\left(p+1\right)-n_{Nlow}p.$$

where *nint*() denotes the nearest integer. This under-determined system can be solved because the Nyquist numbers are constrained integers (see [11] for details).

Then, the unwrapped velocity values can be computed for each VENC:

(5) $$\begin{array}{c} Vu_{high}=Vm_{high}+2n_{Nhigh}\cdot VENC_{high},\;\\ Vu_{low}=Vm_{low}+2n_{Nlow}\cdot VENC_{low} \end{array}$$

Finally, the unambiguous velocity *Vu* is obtained as a weighted mean:

(6) $$Vu=\frac{\left(Vu_{high}+\frac{p}{p+1}Vu_{low}\right)}{1+\frac{p}{p+1}}.$$

In the weighted sum, lower weights are assigned to low VENC data that required more pixels to be corrected.

Of note, this method can be generalized to several VENC values [11]; in this study, however, we used a dual-VENC approach.

To facilitate understanding of the method, let us consider the example of a dual-VENC scheme with a ratio of $\frac{p}{p+1}=\frac{3}{4}$. In this case, the extended velocity dynamic range is three-fold the range of *VENC*_*high*_. From equation (4) and the additional constraints, we obtain seven possible combinations of *n*_*Nhigh*_ and *n*_*Nlow*_ that are given in the lookup Table 1. To unwrap, we calculated the values of equation (4) (Table 1, first column) from which we deduced the two Nyquist numbers (Table 1, second and third column) that allowed us to calculate unambiguous velocities using equation (5). The final result was then obtained using equation (6).

**Table 1:** Look-up table used to compute the Nyquist numbers for a dual-VENC scheme with a ratio of 3/4.

$nint\left(\left(p+1\right)\frac{Vm_{low}-Vm_{high}}{pVENC_{high}}\right)$	*n*_*Nhigh*_	*n*_*Nlow*_
−3	0	1
−2	1	2
−1	−1	−1
0	0	0
1	1	1
2	−1	−2
3	0	−1

*VENC* velocity encoding, *V_m_* measured velocity

#### 2.2.2 Correction

Velocity unwrapping methods fail in the presence of noise and flow acceleration errors in the individual VENC images because the assumption underlying the unwrapping process is no longer valid. Consequently, to mitigate these errors, we proposed an additional correction to the velocity unwrapping algorithm as follows: i) a 3D boxcar mean filter with a kernel of size 9 × 9 × 9 was used to compute a mean velocity map; ii) wrongly corrected voxels were defined as the voxels for which the difference between the velocity and the mean filtered velocity was higher than *VENC*_*high*_; for those voxels, the estimated Nyquist number was modified by summing (or subtracting) *nint*(*d*∕*VENC*_*high*_); iii) the corrected Nyquist number map was then used to unwrap and recombine the individual VENC images into the final result. Using a VENC ratio of 2/3, the dynamic range of the final result was 2 ⋅ *VENC*_*high*_. Using a VENC ratio of 3/4, the dynamic range of the final result was 3 ⋅ *VENC*_*high*_.

The proposed algorithm will be henceforth referred to as coprime dual-VENC (*coDV*).

#### 2.2.3 Standard dual-VENC unwrapping

A standard dual-VENC based velocity unwrapping algorithm [6] was implemented for comparison with the proposed algorithm. Specifically, standard velocity unwrapping consisted in computing the number of phase wraps *n* present in the *VENC*_*low*_ using the *VENC*_*high*_ acquisition. Velocity unwrapping was then performed in the identified voxels by adding or subtracting *n* ⋅ *VENC*_*low*_. In the paper, we refer to the standard unwrapping algorithm as standard dual-VENC (*sDV*).

### 2.3 Data acquisitions

#### 2.3.1 In vitro experiments

**Fig. 1:**
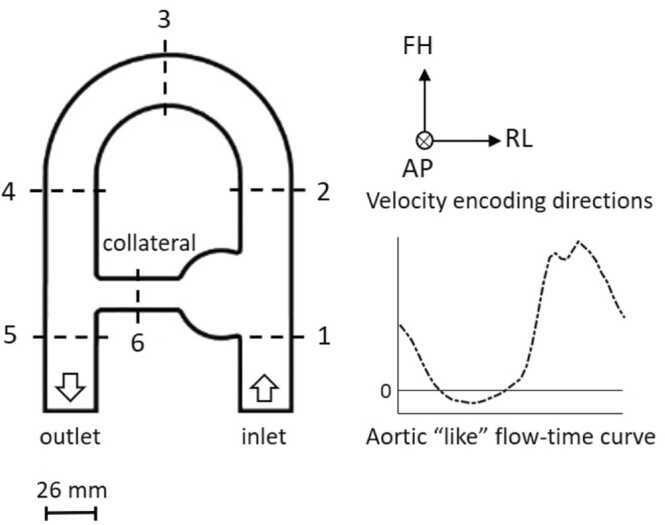
Schematic representation of the coronal view of the phantom, including velocity encoding directions and the inlet flow-time curve

To test and validate the dual-VENC sequence and unwrapping algorithm, we acquired *in vitro* data using a pulsatile flow phantom [18] designed to reproduce complex flow patterns similar to those found in the cardiovascular system. A schematic representation of the phantom geometry is given in Fig. 1. The phantom was made of Nylon and embedded in a silicone bath to increase the signal-to-noise ratio (SNR) and connected to a programmable pump (CardioFlow 5000 MR, Shelley Medical Imaging Technologies, London, Ontario, Canada) installed outside the 5 Gauss line *via* pipes. The pump’s pulsatile flow rate was measured using an ultrasonic flowmeter (UF25B100 Cynergy3 components Ltd, Wimborne, Dorset, UK) placed upstream of the phantom’s entrance. The flow-time curve was chosen to represent an idealized *in vivo* aortic input function (Fig. 1). A blood-mimicking fluid with a kinematic viscosity of *ν* = 4.0210^−6^ *m*^2^∕*s* and a density of *ρ* = 1020 *kg*∕*m*^3^ was supplied to the phantom circuit.

CMR was performed on a 1.5T Ingenia MR system (Philips Healthcare, Best, The Netherlands) using a 32-element torso coil. We acquired three dual-VENC sequences (*VENC*_*low*_ = 27 cm/s, *VENC*_*high*_ = 40 cm/s), (*VENC*_*low*_ = 30 cm/s, *VENC*_*high*_ = 40 cm/s) and (*VENC*_*low*_ = 40 cm/s, *VENC*_*high*_ = 60 cm/s) and six single-VENC sequences (VENC = 27, 30, 40, 60, 80 and 120 cm/s) for comparison. The VENC values of the first two dual-VENC acquisitions (27 and 40 cm/s, 30 and 40 cm/s) were selected such that velocity aliasing would be present in both individual VENC images. In addition, two of the dual-VENC acquisitions had a VENC ratio of $\frac{2}{3}$ and the third dual-VENC acquisition had a ratio of $\frac{3}{4}$. A summary of the TR, TE and VENC values for all acquisitions are given in Table 2. All other acquisition parameters were the same for all sequences as follows: flip angle (FA) 8^∘^, parallel imaging sensitivity encoding (SENSE) factors: 1 × 1.5 × 2, 2 mm isotropic acquired voxel, reconstructed to 1.8 × 1.8 × 1.9 - 1.9 × 1.9 × 2 mm^3^.

**Table 2:** Summary of VENC, TR, and TE values for the *in vitro* 4D Flow acquisitions.

Field strength	VENC (cm/s)	TR (ms)	TE (ms)
1.5 T	SV 27	7.2	4.7
	SV 30	7.1	4.6
	SV 40	6.7	4.2
	SV 60	6.3	3.8
	SV 80	6.0	3.5
	SV 120	5.7	3.2
	DV 27, 40	7.2	4.7
	DV 30, 40	7.1	4.6
	DV 40, 60	6.7	4.2

*VENC* velocity encoded, *SV* single-VENC*, DV* dual-VENC, *TR* repetition time*, TE* echo time, *4D* four-dimensional

Similarly, *in-vitro* acquisitions were performed at 3T to assess performance at a second field strength. Acquisition parameters and results are presented as Appendix A, Appendix A.

#### 2.3.2 In silico experiments

To assess the quality of the obtained velocity fields in terms of fidelity to theoretical velocity values, CFD simulations were carried out to represent the flow in the phantom using the previously published YALES2BIO simulation solver [18], [19].

For a straight forward comparison, the CFD velocity fields underwent phase-averaging and spatial downsampling as described in [18]. Specifically, the phase-averaged velocity $\overset{¯}{u}\left(x,t\right)$ at spatial coordinates **x **= (x,y,z) and at time *t* was defined as follows:

(7) $$\overset{¯}{u}\left(x,t\right)=\frac{1}{N}\overset{N-1}{\underset{k=0}{\sum }}u\left(x,t+kT\right)$$

where **u**(**x**,t) refers to instantaneous velocity vector and N is the total number of cycles of period T. Phase-averaging the CFD velocity field removed the cycle-to-cycle fluctuations that occurred when simulating such an unsteady flow. This process reflects the 4D flow MR measurement that is performed over numerous cardiac cycles.

The resulting phase-averaged CFD velocity field was then registered to the 4D Flow volume and downsampled to the same spatial resolution. Downsampling involved linear interpolation of the CFD velocity from the nodes of the unstructured grid to a high spatial resolution structured grid followed by averaging of neighboring values to obtain a grid with the MRI spatial resolution.

#### 2.3.3 In vivo experiments

Three patients, presenting with aortic dissection (male, age 87), bicuspid aortic valve (BAV) (female, age 75), and Turner syndrome (female, age 36), underwent imaging of the thoracic aorta using the 1.5T MRI system. The imaging protocol included a single-VENC (VENC = 200 − 220 cm/s) and a dual-VENC 4D Flow acquisition with a VENC ratio of 2/3, i.e. *VENC*_*low*_ = 67 − 73 cm/s and *VENC*_*high*_ = 100 − 110 cm/s. Prospective respiratory gating was performed using pencil beam navigator. Sequence parameters for the single-VENC were repetition time (TR) = 4.8-4.9 ms, TE = 2.7-2.8 ms, FA = 5-8^∘^, acquired spatial resolution 2.5 × 2.5 × 2.5 − 2.8 × 2.8 × 2.8*mm*^3^, parallel imaging SENSE factors: 1 × 1.4 × 1.8 - 1 × 1.6 × 2. Sequence parameters for the dual-VENC were: TR = 5.6-5.7 ms, TE = 3.5-3.6 ms, FA = 5-8^∘^, acquired spatial resolution 2.7 × 2.7 × 2.5 - 2.9 × 2.9 × 2.8 mm^3^, parallel imaging SENSE factors (M × P × S): 1 × 1.4 × 1.8 - 1 × 1.8 × 2.1.

### 2.4 Data analysis

#### 2.4.1 Image reconstruction and pre-processing

Raw k-space data were exported for all 4D flow acquisitions. Image reconstruction of each individual velocity encoding was performed offline using a SENSE reconstruction algorithm implemented in ReconFrame (GyroTools, Zürich, Switzerland). Velocity encodings were then combined using complex phase subtraction to obtain the corresponding velocity maps. Eddy current correction was performed on all individual velocity maps using a second-order polynomial fit of the static part of the phantom on a slice-by-slice basis. The mask of the static part of the phantom, corresponding to the silicone bath surrounding the flow region, was obtained by thresholding the magnitude image of the single-VENC 40 acquisition. For the same acquisition, phase contrast angiography (PCMRA) was computed using the absolute values of the velocity vectors weighted by the magnitude values and averaged over time. The PCMRA was used to segment the flow region of the phantom. Subsequently, the generated masks of the static region and the flow region were used in the analysis of all acquired *in vitro* datasets. Similarly, for *in vivo* exams, the static tissue mask was obtained by thresholding the magnitude image of the single-VENC acquisition and the aorta and heart masks were obtained by semi-automatic segmentation of the computed time averaged PCMRA. Segmentation was performed using a MATLAB-based finite element analysis toolbox dedicated to 4D Flow analysis in the aorta [20].

#### 2.4.2 Velocity unwrapping and performance assessment

The proposed coDV algorithm described above was used for all acquired dual-VENC acquisitions. To assess the performance of the unwrapping algorithms on datasets with the same velocity dynamic range, sDV based velocity unwrapping was performed on datasets composed of individual VENC acquisitions with the *VENC*_*high*_ having the same dynamic range as the result of the coDV algorithm.

Qualitative assessment of velocity unwrapping performance was performed by visual inspection of the de-aliased datasets for each individual velocity encoding direction. For the *in vitro* acquisitions, the ability of coDV to reconstruct velocity maps was verified by a visual comparison with the results of CFD simulations, which served as the ground truth. For the *in vivo* acquisitions, the velocity fields were interpolated onto a tetrahedral mesh computed for the segmented flow domain using the aforementioned Matlab-based toolbox, and the results were used to compute streamlines in Paraview (Kitware, Clifton Park, New York ) to qualitatively assess the performance of the unwrapping algorithms.

Quantitative assessment was performed by computing time-resolved average velocity curves and velocity-to-noise ratios (VNR) in six cross-sections positioned orthogonal to the main conduit and to the collateral for the *in vitro* acquisitions (Fig. 1) and ten cross-sections orthogonal to the thoracic aorta for the *in vivo* acquisitions. VNR was computed on speed images as the ratio between the average speed in the cross-section and the standard deviation of noise in the static tissue mask. In addition, on the *in-vitro* acquisitions, the number of pixels with velocity aliasing after unwrapping were manually counted for each dataset in each of the three velocity encoding directions. To assess consistency with the underlying flow physics, the velocity field divergence was computed for the CFD (downsampled and phase-averaged) and for the *in vitro* SV, sDV, and coDV datasets. In analogy with the cardiac cycle, the instants of maximum and minimum flow are referred to as peak systole and diastole, respectively.

## 3 Results

All single- and dual-VENC 4D flow acquisitions were successfully reconstructed and velocity unwrapping was performed using the two methods as described above.

**Fig. 2:**
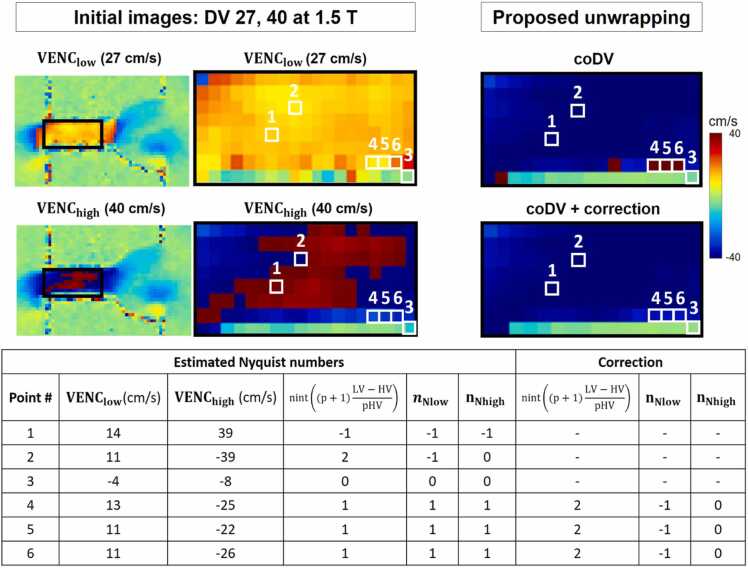
Illustration of the proposed coDV method in the collateral tube of the flow phantom at peak systole. Initial low-VENC and high-VENC velocity maps encoded in the right-left direction are presented on the left: *VENC*_*low*_ = 27 cm/s (top) and *VENC*_*high*_ = 40 cm/s (bottom); the results of the proposed unwrapping are presented on the right: before (top) and after correction (bottom). The table shows the velocity values, estimated Nyquist numbers, and corrected Nyquist numbers for six highlighted pixels. Pixels 1, 2, and 3 exemplify accurately estimated Nyquist numbers. Conversely, pixels 4, 5, and 6 demonstrate incorrect Nyquist number estimations attributable to noise, resulting in errors in the first step of the algorithm. The correction step successfully rectifies the Nyquist numbers for these pixels. To be noted that the proposed method successfully corrects not only isolated pixels but also interconnected ones (4,5 and 6 in the presented example). *VENC* velocity encoding parameter, *coDV* coprime dual-VENC

To illustrate the proposed coDV method, we highlighted the collateral tube region, in which both *VENC*_*low*_ and *VENC*_*high*_ velocity maps presented velocity aliasing, in Fig. 2. Instances where velocity estimates were erroneous due to noise are highlighted. The correction step effectively addressed these errors, demonstrating its efficacy in correcting both isolated and interconnected regions.

### 3.1 *In vitro* unwrapping performance

**Fig. 3:**
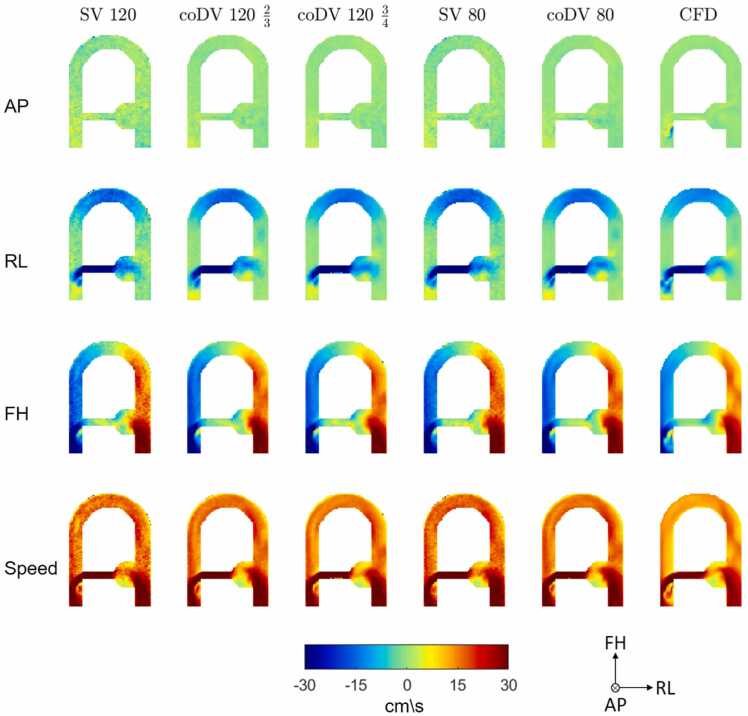
Coronal slice through the middle of the flow phantom presenting velocity maps in the AP (first row), RL (second row) and FH (third row) encoding directions, and velocity magnitude (speed, bottom row). Velocity maps obtained with the proposed coDV method and with SV acquisition show good agreement with CFD (right column) for two different velocity dynamic ranges (80 and 120 cm/s). *AP* anterior-posterior, *FH* feet-head, *RL* right-left, *coDV* coprime dual-VENC, *SV* single-VENC, *CFD* computational fluid dynamics

Qualitative comparisons between the velocity maps for the three encoding directions obtained with single-VENC, coDV, and CFD, at peak systole, are presented in Fig. 3. Flow patterns appeared consistent between velocity dynamic ranges (Fig. 3) and field strengths (Appendix A, Appendix A). The right-left and foot-head (FH) velocity maps revealed excellent agreement with the CFD, showing highly consistent velocity patterns even in complex flow regions. The anterior-posterior (AP) component showed some discrepancies, particularly for the higher dynamic range (120 cm/s) in the single-VENC, which was expected given the low velocity amplitudes in this encoding direction, as also reported in [18].

Table 3  reports the number of aliased pixels in individual velocity maps for the results of sDV and coDV. It is noteworthy that, irrespective of velocity dynamic range and VENC ratio, the proposed coDV method demonstrated superior performance compared to the sDV.

**Table 3:** Number of pixels with velocity aliasing counted in result images of sDV and proposed unwrapping (coDV) for different VENC ratios.

Reconstructed dynamic range	Individual velocity images	Unwrapping method	VENC ratio	Number of pixels with velocity aliasing			
				RL	AP	FH	Total
80 cm/s	DV 27, 40	coDV	2/3	2	0	1	3
	SV 27 and SV 80	sDV		30	0	10	40
120 cm/s	DV 40, 60	coDV	2/3	0	0	0	0
	SV 40 and SV 120	sDV		6	10	37	53
120 cm/s	DV 30, 40	coDV	3/4	7	0	8	15
	SV 30 and SV 120	sDV		13	3	6	22

*sDV* standard unwrapping*, VENC* velocity encoded*, coDV* coprime dual-VENC

**Fig. 4:**
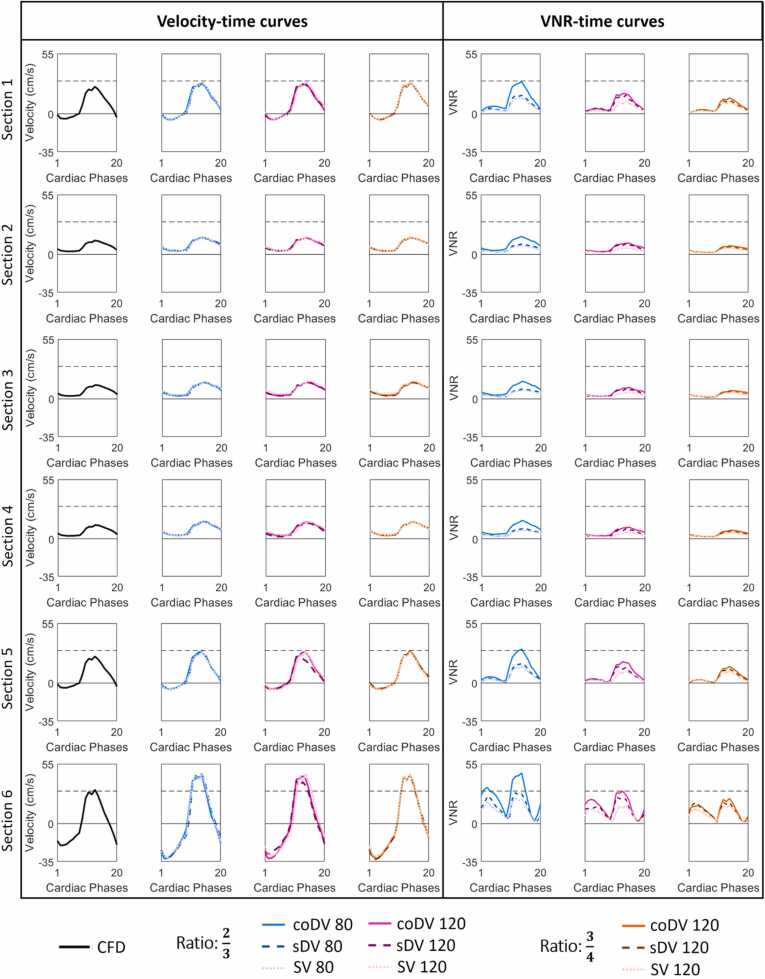
Velocity time curves and VNR time curves for different velocity dynamic ranges measured in six representative cross-sections. Good agreement can be observed between the SV acquisitions and the results of de-aliasing algorithms sDV and coDV for all the datasets and CFD simulations. *VNR* velocity-to-noise ratio, *SV* single-VENC, *sDV* standard dual-VENC, *coDV* coprime dual-VENC, *CFD* computational fluid dynamics

Time resolved velocity and VNR curves for the six cross-sections are presented in Fig. 4. Note that the measured velocity time-curves were generally similar to the CFD simulated time-curves. In section 6, however, the measured maximum velocities were higher than those expected by the CFD simulation. This discrepancy was likely due to the acceleration of flow in the collateral tube, a known source of velocity quantification error. Higher VNR values have been obtained for the coDV method in comparison to both single-VENC and standard dual-VENC. In particular, average VNR gains across sections obtained for coDV respectively for diastole and systole were 80 ± 20% and 110 ± 3% with respect to SV and 70 ± 5% and 75 ± 3% with respect to sDV.

Table 4 presents the mean and standard deviation, with corresponding 95% confidence intervals, of the velocity field divergence computed for the downsampled CFD and *in vitro* measurements for the different velocity encodings and unwrapping methods. To assess whether these differences were statistically significant, Levene’s test [21] was performed, confirming variance inequality among SV, sDV, and coDV (*p*<0.05).

**Table 4:** Mean and standard deviation, with corresponding 95% confidence intervals, of the divergence (1/s) computed for downsampled CFD and *in vitro* measured velocity fields.

Velocity field	Mean of Divergence	Mean CI (95%)	Std Dev of Divergence	Std Dev CI (95%)
CFD	−0.01	[−0.04, 0.00]	6.65	[6.63, 6.66]
coDV 80	−0.20	[−0.22, −0.18]	7.35	[7.33, 7.37]
sDV 80	−0.30	[−0.33, −0.28]	7.53	[7.51, 7.55]
SV 80	−0.32	[−0.36, −0.29]	11.15	[11.12, 11.17]
coDV 120 (2/3)	−0.23	[−0.26, −0.21]	8.12	[8.10, 8.14]
sDV 120 (2/3)	−0.15	[−0.18, −0.13]	8.40	[8.38, 8.42]
coDV 120 (3/4)	−0.24	[−0.27, −0.21]	7.84	[7.82, 7.86]
sDV 120 (3/4)	−0.28	[−0.30, −0.25]	7.58	[7.57, 7.60]
SV 120	−0.34	[−0.39, −0.29]	14.55	[14.51, 14.58]

*CFD* computational fluid dynamics*, CI* confidence interval*, coDV* coprime dual-VENC*, sDV* standard dual-VENC*, SV* single-VENC

### 3.2 *In vivo* unwrapping performance

**Fig. 5:**
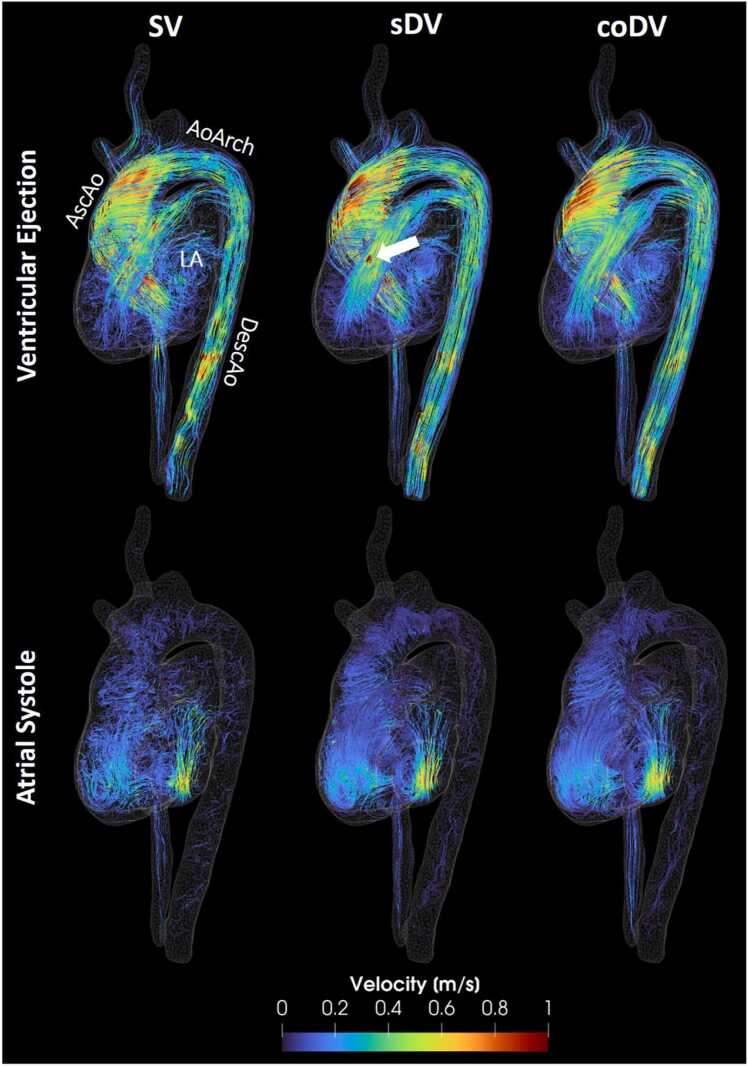
Flow streamlines in the heart and aorta computed at two representative cardiac phases (ventricular ejection and atrial systole) for the three different datasets: SV, standard unwrapping (sDV), and proposed unwrapping (coDV). The white arrow points at residual aliasing in sDV. *AscAo* ascending aorta, *AoArch* aortic arch, *DescAo* descending aorta, *LA* left atrium, *SV* single-VENC, *sDV* standard dual-VENC

**Fig. 6:**
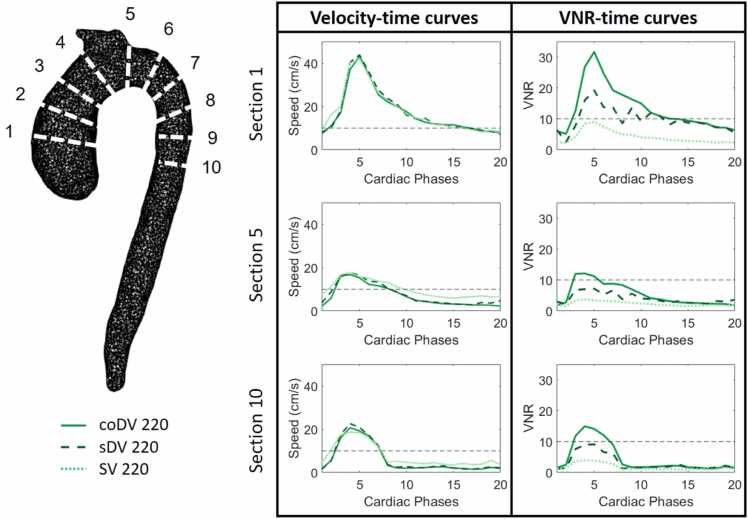
Velocity time curves and VNR time curves for coDV, sDV and SV in the BAV patient in three cross-sections along the aorta. *BAV* bicuspid aortic valve. *VNR* velocity-to-noise ratio, *coDV* coprime dual-VENC, *sDV* standard dual-VENC, *SV* single-VENC, *BAV* bicuspid aortic valve

Fig. 5  shows flow streamlines in the aorta and heart computed for SV, sDV, and coDV at two representative cardiac phases for the BAV patient. As expected, the single-VENC acquisition exhibits the strongest noise level, irrespective of the cardiac phase. Regarding the dual-VENC based velocity unwrapping, residual velocity aliasing can clearly be observed in the sDV results (white arrow) while absent in the coDV result. Fig. 6 shows velocity and VNR time curves for coDV, sDV, and SV in three cross-sections along the aorta. In this instance, similar to the *in vitro* results, coDV demonstrated higher performance in terms of VNR compared to sDV and SV.

The average VNR gains for the first patient across ten cross-sections (Fig. 6) obtained for coDV were 250 ± 36% and 84 ± 8% in diastole and systole, respectively, with respect to SV, and 158 ± 91% and 20 ± 6% with respect to sDV. For the second patient, the VNR gains for coDV were 111 ± 29% in systole and 180 ± 30% in diastole with respect to SV, and 18 ± 13% in systole and 17 ± 14% in diastole with respect to sDV. For the third patient, the VNR gains for coDV were 345 ± 35% and 335 ± 77% in systole and diastole with respect to SV, and 31 ± 10% and 21 ± 22% in systole and diastole with respect to sDV.

## 4 Discussion

We developed and validated a dual-VENC 4D Flow CMR sequence with coprime VENC ratios that extended the measurement velocity dynamic range. In addition, we proposed a dedicated time efficient velocity unwrapping algorithm. We demonstrated improved performance of our method compared to standard dual-VENC based velocity unwrapping in a pulsatile flow phantom. Finally, we showed clinical feasibility in patients with pathologies of the aorta.

The choice of the VENC value in single-VENC 2D and 4D Flow MRI represents a fundamental trade-off between VNR and velocity dynamic range. Standard dual-VENC MRI involves taking two measurements with different velocity limits—*VENC*_*low*_ and *VENC*_*high*_. The result of the combination of the two has the good VNR of the *VENC*_*low*_ measurement and the high dynamic range of the *VENC*_*high*_ measurement. However, in cases when the exact velocity range to be measured is unknown, *i.e*. in most pathology cases, standard dual-VENC fails if the velocity limit of the *VENC*_*high*_ is insufficient, as velocity aliasing is still be present. It is advantageous then to further extend the unambiguous velocity dynamic range beyond *VENC*_*high*_. Studies in weather radar signal processing [7] and later in color Doppler electrocardiography [11] demonstrated that this can be achieved by setting the velocity limits of the individual measurements closer to each other. Specifically, by using coprime integers to define the ratio between the low and the high velocity limit, in the form of *p*∕(*p* + 1), with *p*≥2, where *p* is the extension factor governing the increase in the extended range.

The proof of concept for 2D one-directional velocity measurements in MRI was presented by [16] using sequential acquisitions. In their approach, the single-VENC velocity estimation was modeled as a least squares periodic function, with the period determined by the VENC value. For dual-VENC velocity estimation, the authors minimized a combined periodic function, incorporating both individual single-VENC functions. The period of this combined function was based on the least common multiplier of the two single-VENC periods. The study demonstrated a significant reduction in aliasing artifacts using ratios of 2/3 and 3/4, although this was achieved through a computationally intensive minimization process.

In this work, we implemented a dual-VENC 4D Flow MRI sequence with extension factors *p*≥2. A computationally costly minimization based approach for velocity unwrapping such as proposed by [16] may prove challenging in the context of 4D Flow MRI given the significantly larger data size of these acquisitions. Similar to Posada et al., we proposed a look up table based velocity unwrapping that is straightforward and computationally efficient. First, the number of wraps is estimated for each individual VENC image, then the final result is computed as a weighted mean of the two unwrapped individual VENC images (*VENC*_*low*_ and *VENC*_*high*_). We demonstrate that VENC ratios of 2/3 and 3/4 enable velocity aliasing correction also in 4D Flow acquisitions, even when both individual VENC acquisitions are aliased. This is a very important advantage of the proposed dual-VENC, because 4D Flow acquisitions span large volumes with potentially complex flow patterns and wide velocity dynamic ranges. Unexpectedly high velocities, unknown prior to acquisition, for example in the presence of stenosis, may lead to aliasing in the *VENC*_*high*_ dataset, context in which standard dual-VENC unwrapping fails [12]. Additionally, our approach is also 100% acquisition efficient, since there is no data rejection. The weighted mean combination reduces the noise in the resulting image, further improving VNR, compared to standard dual-VENC. In addition, we demonstrate improved consistency with the flow physics by the significant reduction in the variance of the velocity field divergence for dual-VENC compared to single-VENC datasets.

Use of extension factors of 2, 3, or 4 is recommended to balance velocity range and measurement quality [10] as higher extension factors can degrade measurement accuracy, due to reduced robustness to noise. In line with previous studies, our findings demonstrate, in a very controlled *in vitro* setting, that a VENC ratio of 2/3 outperforms a ratio of 3/4 in terms of final numbers of uncorrected pixels. Nevertheless, it is important to note that the 3/4 ratio enables further decrease in the VENC values used to obtain the same extended dynamic range as a 2/3 ratio. Improving measurements of low velocities by lowering VENC is especially important in the context of 4D Flow imaging, as discussed above, since the large volume coverage leads to simultaneous measures of fast arterial and slow venous flows. In single-VENC 4D Flow MRI, slow flow regions have much lower VNR and the measurement sensitivity is inherently lower, leading to decreased measurement precision [22].

PC MRI is based on the assumption that the velocity to be measured is constant during the application of the velocity encoding gradients. Consequently, both single- and dual-VENC approaches result in measurement errors due to acceleration and turbulence [12]. Following the proof of concept of high VENC ratio dual-VENC for 2D Flow MRI, Franco et. al. investigated the robustness of the method to measurement errors and proposed a dedicated correction algorithm that further improved velocity unwrapping performance [17]. Similarly, we addressed the sensitivity of the proposed method to measurement errors by adding a patch based correction step, to identify aliased pixels and re-estimate the number of wraps to be corrected. We demonstrated efficacy in correcting larger regions of aliasing in addition to isolated pixels. The patch size was determined empirically based on the *in vitro* dataset, and may not be optimal for applications *in vivo*. Advanced velocity field denoising combining smoothing and enforcing mass conservation will likely further improve performance in noisy regions.

As anticipated from the *in vitro* results, the *in vivo* datasets demonstrated improved VNR. This improvement is evident in the streamline visualizations of the BAV patient, which show a reduction in noise achieved through the implementation of the proposed method. VNR analysis along the aortic centerline confirmed that the significant gains observed in coDV compared to sDV and single-VENC were also attainable *in vivo*. These results were achieved despite inherent challenges such as potential cardiac rhythm variations and subject motion. For this study, we developed a dual-VENC sequence with eight velocity encodes. In theory, only seven encodes would be required, as the reference segment could be shared to save acquisition time. However, the 7-point approach only allows for asymmetric velocity encoding. Hadamard velocity encoding, on the other hand, results in shorter TRs, shorter scan time, and improved temporal resolution, but it can only be used with an 8-point scheme [13]. It could be mentioned that significant efforts have been made to correct wrapped velocities in single-VENC acquisitions [23], [24], [25]. While these techniques do not suffer from the cost of increased acquisition time, they may fail in the presence of significant velocity jumps when the chosen VENC is too low. Therefore, the achievable VNR appears limited compared to dual-VENC. Also, they may come at the cost of increased computation time, that may not be compatible with an online use at the scanner.

## 5 Limitations

In the present study, the maximum achievable velocity in the phantom was comparable to the venous flow velocities typically observed in humans. To test the algorithm and ensure aliasing occurred in both *VENC*_*low*_ and *VENC*_*high*_, we performed acquisitions using relatively low VENC values. This approach led to longer echo times (TEs), which could introduce errors associated with higher-order motion encoding [26]. Despite this, we observed strong agreement between the measured velocity fields and the numerical values obtained through CFD.

The proposed method theoretically allows for the selection of low VENCs to achieve high VNRs. However, the truth of this statement is limited by the presence of errors in the *VENC*_*low*_ images. The *VENC*_*low*_ image is acquired with a large first moment, which requires the use of large flow-encoding gradients. This increases the image’s sensitivity to intra-voxel dephasing, acceleration errors, and signal loss due to turbulent or unsteady flow [27]. This can result in errors in regions with high velocity dispersion, such as near vessel walls or distal to a stenosis. In addition, an interleaved implementation in the dual-VENC sequence, forces the *VENC*_*high*_ images to be performed in the same TR of *VENC*_*low*_. However, a recent consensus statement on 4D flow [28] suggests that interleaved velocity encoding is preferred to avoid inter-cycle variability.

Another potential limitation is the increase in acquisition time, which is however inherent to all dual-VENC techniques. In further studies it will be important to investigate k-space undersampling techniques that would allow dual-VENC sequences to be performed at comparable scan times with single-VENC ones, and benefit from the significant increase in VNR to mitigate undersampling induced noise.

## 6 Conclusions

We proposed and validated a 4D Flow dual-VENC sequence that uses extension factors *p*≥2. Good performance of the proposed aliasing correction method and significant VNR improvement was demonstrated *in vitro* at two different field strengths. A CFD simulation of the flow within the phantom was performed for comparison. The resulting flow parameters were found to be in accordance with the velocity field obtained utilizing the proposed methodology. Our *in vitro* results suggest that a ratio of 2/3 may outperform a ratio of 3/4 in terms of VNR gains. In addition, we demonstrated *in vivo* feasibility in different vascular pathologies.

## Author contributions

**Philippe Douek:** Resources. **Damien Garcia:** Writing – review & editing, Methodology. **Monica Sigovan:** Writing – original draft, Validation, Supervision, Resources, Formal analysis, Data curation, Conceptualization. **Marta Beghella Bartoli:** Writing – original draft, Visualization, Software, Investigation, Formal analysis, Data curation. **Sara Boccalini:** Resources. **David Chechin:** Resources. **Loic Boussel:** Resources, Conceptualization.

## Declaration of competing interests

The authors declare that they have no known competing financial interests or personal relationships that could have appeared to influence the work reported in this paper.

## Footnotes

Appendix A Supplementary data associated with this article can be found in the online version at doi:10.1016/j.jocmr.2025.101871.
