# Supplementary material for: Coprime dual-velocity encoding for extended velocity dynamic range in 4D flow magnetic resonance imaging
Source: J Cardiovasc Magn Reson. 2025 Mar 7;27(1):101871. doi: 10.1016/j.jocmr.2025.101871 (PMC12032882; doi:10.1016/j.jocmr.2025.101871)
Supplement: Supplementary file 1 — Supplementary material [file mmc1.pdf]

# Supplementary Materials: Coprime dual-VENC for extended velocity dynamic range in 4D Flow MRI

Marta Beghella Bartoli[a], Sara Boccalini[a,b], David Chechin[c], Loic Boussel[a,b], Philippe Douek[a,b], Damien Garcia[a], Monica Sigovan[a]

[a] *University of Lyon, CREATIS Laboratory, Lyon, France*
[b] *Department of Radiology, Hospices Civils de Lyon, Lyon, France*
[c] *Philips, Surenes, France*

**Abstract**

This document provides additional details and analyses supporting the results presented in the main manuscript titled *Coprime dual-VENC for extended velocity dynamic range in 4D Flow MRI*.

## *0.1. Data acquisition*

### *0.1.1. In vitro experiments*

MR imaging was performed on a 3 T Ingenia Elition X system (Philips, Best, The Netherlands) using a 32 elements torso coil. At 3 T, we acquired two dual-VENC sequences ($VENC_{low}$ = 27 cm/s, $VENC_{high}$ = 40 cm/s) and ($VENC_{low}$ = 40 cm/s, $VENC_{high}$ = 60 cm/s), both with a VENC ratio of $\frac{2}{3}$, and four single-VENC acquisitions (VENC = 27, 40, 80, and 120 cm/s). A summary of the TR, TE and VENC values for 3T acquisitions are given in 0.1.1. All other acquisition parameters were the same: FA 8°, parallel imaging SENSE factors: $1 \times 1.5 \times 2$, 2 mm isotropic acquired voxel, reconstructed to $1.8 \times 1.8 \times 1.9$ - $1.9 \times 1.9 \times 2$ $mm^3$.

## *0.2. In vitro unwrapping performance*

Qualitative comparisons of velocity maps for the three encoding directions between the single-VENC, coDV, and CFD, at peak systole, are presented in figure 1. Flow patterns appear consistent between field strengths and velocity dynamic ranges. The RL and FH velocity maps reveal excellent agreement with the CFD, showing highly consistent velocity patterns even in complex flow regions. The AP component shows some discrepancies, particularly

| VENC (cm/s) | TR (ms) | TE (ms) |
|---|---|---|
| SV 27 | 9.8 | 5.6 |
| SV 40 | 9 | 4.9 |
| SV 80 | 8.1 | 4.2 |
| SV 120 | 5.7 | 3.2 |
| DV 27,40 | 9.8 | 5.6 |
| DV 40,60 | 6.7 | 4.2 |

Table 1: Summary of VENC, TR, and TE values for the *in vitro* 4D Flow acquisitions at 3 T.

| **Reconstructed dynamic range** | **Individual velocity images** | **Unwrapping method** | **Ratio $\frac{p}{q}$** | **Number of pixels with velocity aliasing** | | | |
|---|---|---|---|---|---|---|---|
| | | | | **RL** | **AP** | **FH** | **Total** |
| 80 cm/s | DV 27, 40 | coDV | 2/3 | 37 | 2 | 3 | 42 |
| | SV 27 and SV 80 | sDV | | 5 | 12 | 28 | 45 |
| 120 cm/s | DV 40, 60 | coDV | 2/3 | 0 | 5 | 1 | 6 |
| | SV 40 and SV 120 | sDV | | 5 | 3 | 14 | 22 |

Table 2: Number of pixels with velocity aliasing counted in result images of standard unwrapping (sDV) and proposed unwrapping (coDV) at 3 T for different VENC ratios.

for the higher dynamic range (120 cm/s) in the single-VENC, which was expected given the low velocity amplitudes in this encoding direction.

Table 2 reports the number of aliased pixels in individual velocity maps for the results of sDV and coDV at 3 T. It is noteworthy that, irrespective of field strength, velocity dynamic range the proposed coDV method demonstrated consistently superior performance compared to the sDV.

Time resolved velocity and VNR curves for the six cross-sections are presented in Figure 2. Note that the measured velocity time-curves were generally similar to the CFD simulated time-curves. In section 6 however, the measured maximum velocities were higher than those expected by the CFD simulation. This discrepancy was likely due to the acceleration of flow in the collateral tube, a known source of velocity quantification error. Higher VNR values have been obtained for the coDV method in comparison to both single-VENC and standard dual-VENC.

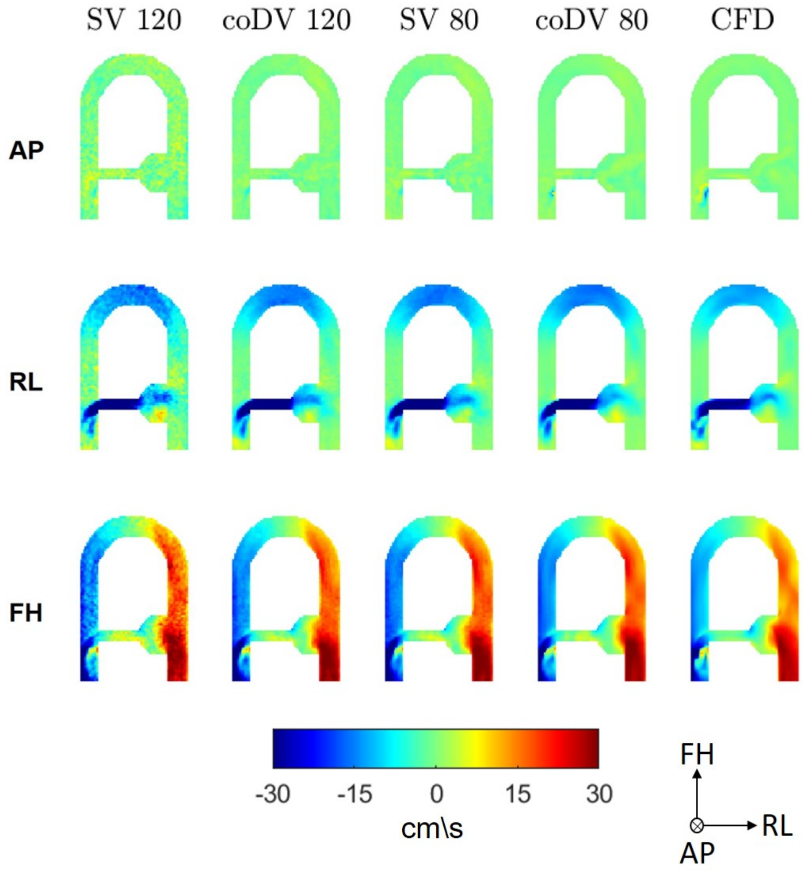


Figure 1: Coronal slice through the middle of the flow phantom presenting velocity maps in the AP (top), RL (middle) and FH (bottom) encoding directions. Velocity maps obtained with the proposed coDV method and with SV acquisition show good agreement with CFD (right column) for two different velocity dynamic ranges (80 and 120 cm/s) at 3 T.

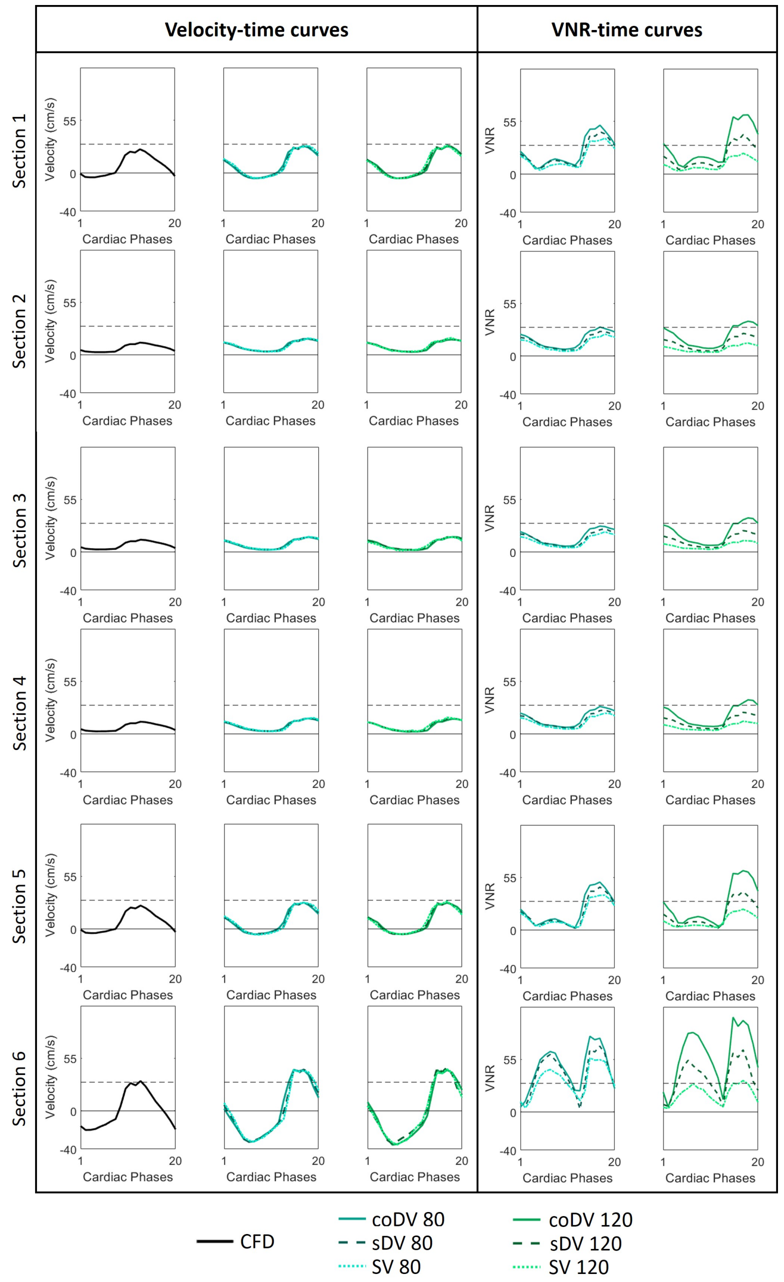


Figure 2: Velocity time curves and VNR time curves for different velocity dynamic ranges measured in six representative cross-sections. Good agreement can be observed between the SV acquisitions and the results of de-aliasing algorithms sDV and coDV for all the datasets and CFD simulations.
